# Cardiac protective effects of irbesartan via the PPAR-gamma signaling pathway in angiotensin-converting enzyme 2-deficient mice

**DOI:** 10.1186/1479-5876-11-229

**Published:** 2013-09-25

**Authors:** Zhen-Zhou Zhang, Qian-Hui Shang, Hai-Yan Jin, Bei Song, Gavin Y Oudit, Lin Lu, Tong Zhou, Ying-Le Xu, Ping-Jin Gao, Ding-Liang Zhu, Josef M Penninger, Jiu-Chang Zhong

**Affiliations:** 1 State Key Laboratory of Medical Genomics, Ruijin Hospital Affiliated to Shanghai Jiao Tong University School of Medicine, Shanghai Institute of Hypertension, 197 Ruijin 2nd Road, Shanghai, 200025, China; 2 Shanghai Key Laboratory of Hypertension, Shanghai Institute of Hypertension, Shanghai, 200025, China; 3 Department of Cardiology and Institute of Clinical Medicine Research, Affiliated Hospital of Zunyi Medical College, Zunyi, 563003, China; 4 Department of Mental Health, Ruijin Hospital, School of Medicine, Shanghai Jiao Tong University, Shanghai, 200025, China; 5 Divsion of Cardiology, Department of Medicine, University of Alberta, Mazankowski Alberta Heart Institute, Edmonton, T6G 2S2, Canada; 6 Department of Cardiology, Ruijin Hospital, School of Medicine, Shanghai Jiao Tong University, Shanghai, 200025, China; 7 Department of Pediatrics, Ruijin Hospital, School of Medicine, Shanghai Jiao Tong University, Shanghai, 200025, China; 8 Institute of Molecular Biotechnology of the Austrian Academy of Sciences, Vienna, Austria

**Keywords:** Angiotensin-converting enzyme 2, Irbesartan, Peroxisome proliferator-activated receptor-γ, Connective tissue growth factor, Myocardial injury

## Abstract

**Background:**

Angiotensin-converting enzyme 2 (ACE2), a monocarboxypeptidase which metabolizes angiotensin II (Ang II) to generate Ang-(1–7), has been shown to prevent cardiac hypertrophy and injury but the mechanism remains elusive. Irbesartan has the dual actions of angiotensin receptor blockade and peroxisome proliferator-activated receptor-γ (PPARγ) activation. We hypothesized that irbesartan would exert its protective effects on ACE2 deficiency-mediated myocardial fibrosis and cardiac injury via the PPARγ signaling.

**Methods:**

10-week-old ACE2 knockout (ACE2KO; Ace2^-/y^) mice received daily with irbesartan (50 mg/kg) or saline for 2 weeks. The wild-type mice (Ace2^+/y^) were used to the normal controls. We examined changes in myocardial ultrastructure, fibrosis-related genes and pathological signaling by real-time PCR gene array, Western blotting, Masson trichrome staining and transmission electron microscope analyses, respectively.

**Results:**

Compared with the Ace2^+/y^ mice, cardiac expression of PPARα and PPARγ were reduced in Ace2^-/y^ mice and the myocardial collagen volume fraction (CVF) and expression of fibrosis-related genes were increased, including transforming growth factor-β1 (TGFβ1), connective tissue growth factor (CTGF), collagen I and collagen III. Moreover, ACE2 deficiency triggered cardiac hypertrophy, increased myocardial fibrosis and adverse ultrastructure injury in ACE2KO hearts with higher levels of atrial natriuretic factor (ANF) and phosphorylated extracellular signal-regulated kinase 1/2 (ERK1/2), without affecting cardiac systolic function. Intriguingly, treatment with irbesartan significantly reversed ACE2 deficiency-mediated pathological hypertrophy and myocardial fibrosis in Ace2^-/y^ mice linked with enhancement of plasma Ang-(1–7) level and downregulation of AT1 receptor in heart. Consistent with attenuation of myocardial fibrosis and ultrastructure injury, the myocardial CVF and levels of ANF, TGFβ1, CTGF, collagen I, collagen III and phosphorylated ERK1/2 were lower, and expression of PPARγ was higher in ACE2KO mice in response to irbesartan treatment, without affecting cardiac expression of PPARα, PPARδ, β-myosin heavy chain, TGFβ2 and fibronectin.

**Conclusions:**

We conclude that irbesartan prevents ACE2 deficiency-mediated pathological hypertrophy and myocardial fibrosis in ACE2 mutant mice via activation of the PPARγ signaling and suppression of the TGFβ−CTGF−ERK signaling, resulting in attenuation of myocardial injury. Drugs targeting ACE2 and PPARγ represent potential candidates to prevent and treat myocardial injury and related cardiac disorders.

## Background

Through the actions of its main biological peptide, angiotensin (Ang) II, the renin-angiotensin system (RAS) has been implicated in myocardial remodeling and heart failure [1-3]. Myocardial remodeling is thought to be the result of a continuum of cardiac structural and functional alterations within cellular and extracellular phenotype, including cardiac myocyte hypertrophy and the extracellular matrix (ECM) remodeling, which trigger disruption of the normal myocardial structure and increased clinical risk of heart failure [1,2,4]. Ang II plays an essential role in myocardial fibrosis by enhancing activation of transforming growth factor-β (TGFβ) and connective tissue growth factor (CTGF), which may further facilitate the extracellular signal-regulated kinase 1/2 (ERK1/2) signaling [1,5,6]. Angiotensin-converting enzyme 2 (ACE2) is a negative regulator of the RAS and has recently been implicated in pathological hypertrophy and heart failure [2,7,8]. We have previously demonstrated that ACE2 overexpression prevents Ang II-mediated cardiovascular remodeling associated with reduction of TGFβ1 [9-12]. However, the exact roles and mechanisms of the ACE2 involved in myocardial hypertrophy, fibrosis and cardiac injury are largely unknown.

The angiotensin type I (AT1) receptor blocker irbesartan has unique chemical properties that enable it to partially activate the peroxisome proliferator-activated receptor-γ (PPARγ), which is a nuclear receptor controlling cardiovascular hypertrophy and dysfunction [3,8,13]. Transgenic mice with cardiomyocyte-specific expression of PPARγ were protected from lipopolysaccharide-induced cardiac dysfunction [14]. Moreover, PPAR-γ has been reported to be a protective regulator in cardiac injury by preventing activation of the CTGF and ERK signaling [15,16]. Our previous studies have revealed that the level of PPAR-γ was downregulated in hypertension-mediated cardiovascular remodeling and injury associated with reduction of ACE2 expression [3]. However, the relationship between ACE2 gene and the PPARγ signaling remains to be fully elucidated. In this work, we directly assessed the hypothesis that irbesartan would exert its protective effects on ACE2 deficiency-mediated myocardial fibrosis and cardiac injury by the modulation of the PPARγ signaling pathway.

## Methods

### Experimental animals and protocols

Ten-week-old male ACE2 knockout (ACE2KO; Ace2^–/y^) mice were backcrossed into a pure C57BL/6 background and treated with irbesartan (Bristol-Myers Squibb Co., Princeton, NJ) in their drinking water (50 mg.kg^-1^.d^-1^) for 14 days as described previously [9,17]. The wild-type (WT; Ace2^+/y^) mice were used as the normal controls. Plasma Ang II and Ang-(1–7) levels were measured by radio-immunoassay analysis as described previously [12,17]. All experiments were approved and performed in accordance with *the Guide for the Care and Use of Laboratory Animals* published by the US National Institutes of Health (NIH Publication No.85-23, revised 1996), Shanghai Jiao Tong University School of Medicine and the Animal Research Ethics Committee at the Canadian Council on Animal Care.

### Echocardiography and myocardial ultrastructure observation

Transthoracic echocardiography was performed and analyzed with a Vevo 770 highresolution imaging system equipped with a 30-MHz transducer (RMV-707B; VisualSonics) in a blinded manner as described previously [2,18]. For transmission electron microscope analysis, samples of mice left ventricle tissues were immediately cut into small pieces and immersed in 2.5% glutaraldehyde as described previously [3]. The myocardial ultrastructure of mice was observed on a HITACHI-600 electron microscope (Hitachi, Japan).

### RNA extraction and real-time PCR gene array

The cardiac mRNA expression of PPARs and fibrosis-related genes in WT and ACE2KO mice were examined using the real-time PCR gene array (The RT^2^ Profiler™ PCR Array Mouse; http://www.sabiosciences.com/rt_pcr_product/HTML/PAMM-038Z.html). The total RNA was extracted from flash-frozen heart tissue using TRIzol extraction protocol (Invitrogen, CA) and purified using a RNeasy® MinElute™ Cleanup Kit (Qiagen, Valencia, CA). Subsequently, total RNA was reverse transcribed using the SuperScript III Reverse Transcriptase (Invitrogen, CA) and complementary DNA was amplified by PCR using the 2X SuperArray PCR Master Mix (SuperArray Bioscience, Frederick, MD). The Real-time PCR Gene Array was then performed on each sample using The PAMM-038Z RT^2^ Profiler™ PCR Array, according to the Manufacturer’s instructions. Data were analyzed using the ∆∆Ct method and expressed as fold changes of the upregulation or downregulation.

### TaqMan real-time PCR analysis

TaqMan Real-time reverse transcription PCR were used to evaluate the cardiac mRNA levels as described previously [2,18,19]. The primer and probe for atrial natriuretic factor (ANF), β-myosin heavy chain (β-MHC), TGFβ1, and fibronectin (FN1) are listed in Table 1. 18S rRNA was used as an endogenous control. All samples were run in triplicates.

**Table 1:** Primer and probe sequences for TaqMan real-time PCR analysis^*^

**Genes**	**Primer/Probe**	**Sequence (Probe: 5′-FAM- -TAMRA-3′)**
ANF	Forward Primer	5′-GGAGGAGAAGATGCCGGTAGA-3′
	Reverse Primer	5′-GCTTCCTCAGTCTGCTCACTCA-3′
	Probe	5′-TGAGGTCATGCCCCCGCAGG-3′
β-MHC	Forward Primer	5′-GTGCCAAGGGCCTGAATGAG-3′
	Reverse Primer	5′-GCAAAGGCTCCAGGTCTGA-3′
	Probe	5′-ATCTTGTGCTACCCAGCTCTAA-3′
TGF-β1	Forward Primer	5′-CCTGCAAGACCATCGACATG-3′
	Reverse Primer	5′-ACAGGATCTGGCCACGGAT-3′
	Probe	5′-CTGGTGAAACGGAAGCGCATCGAA-3′

^*^Primer/probe mix for 18S and Fibronectin (product #: Mm01256742_m1) were obtained from Applied Biosystems Inc. *ANF* atrial natriuretic factor; *β-MHC* β-myosin heavy chain; *TGF-β1* transforming growth factor-β1.

### Western blot analysis

Western blotting analysis was used to measure protein levels of mice hearts as described previously [11,20]. The primary antibody against ERK1/2 (44/42 kD), phospho-ERK1/2 (44/42 kD), PPARγ (53, 57 kD), PPARα (55 kD), PPARδ (52 kD), CTGF (38 kD), Collagen I (150 kD), Collagen III (70 kD), AT1 (41 kD) and α-tubulin (55 kD) were obtained from Cell Signaling Technology (Beverly, MA), Abgent Biotech Co. (San Diego, CA), Abcam Inc. (Cambridge, MA) and Santa Cruz Biotechnology (Santa Cruz, CA), respectively. Aim proteins were detected by enhanced chemiluminescence using X-O-Mat X-ray film.

### Picro-sirius red and Masson trichrome staining

Picro-sirius red (PSR) and Masson trichrome staining were carried out as previously described [12,17]. Masson trichrome-stained sections were used for assessment of overall tissue architecture and interstitial fibrosis. PSR staining of heart sections were used to assess for myocardial interstitial fibrosis.

### Statistical analysis

Values are expressed as mean ± SEM. All statistical analyses were performed with SPSS software (Version 11.5) either by Student’s *t* test for comparison between two groups or by ANOVA followed by the Student-Newman-Keuls test for multiple-comparison testing as appropriate. A value of *P* < 0.05 was considered to indicate statistically significance.

## Results

### Treatment with irbesartan attenuated ACE2 deficiency-mediated myocardial fibrosis and ultrastructure injury in ACE2KO mice

**Figure 1:**
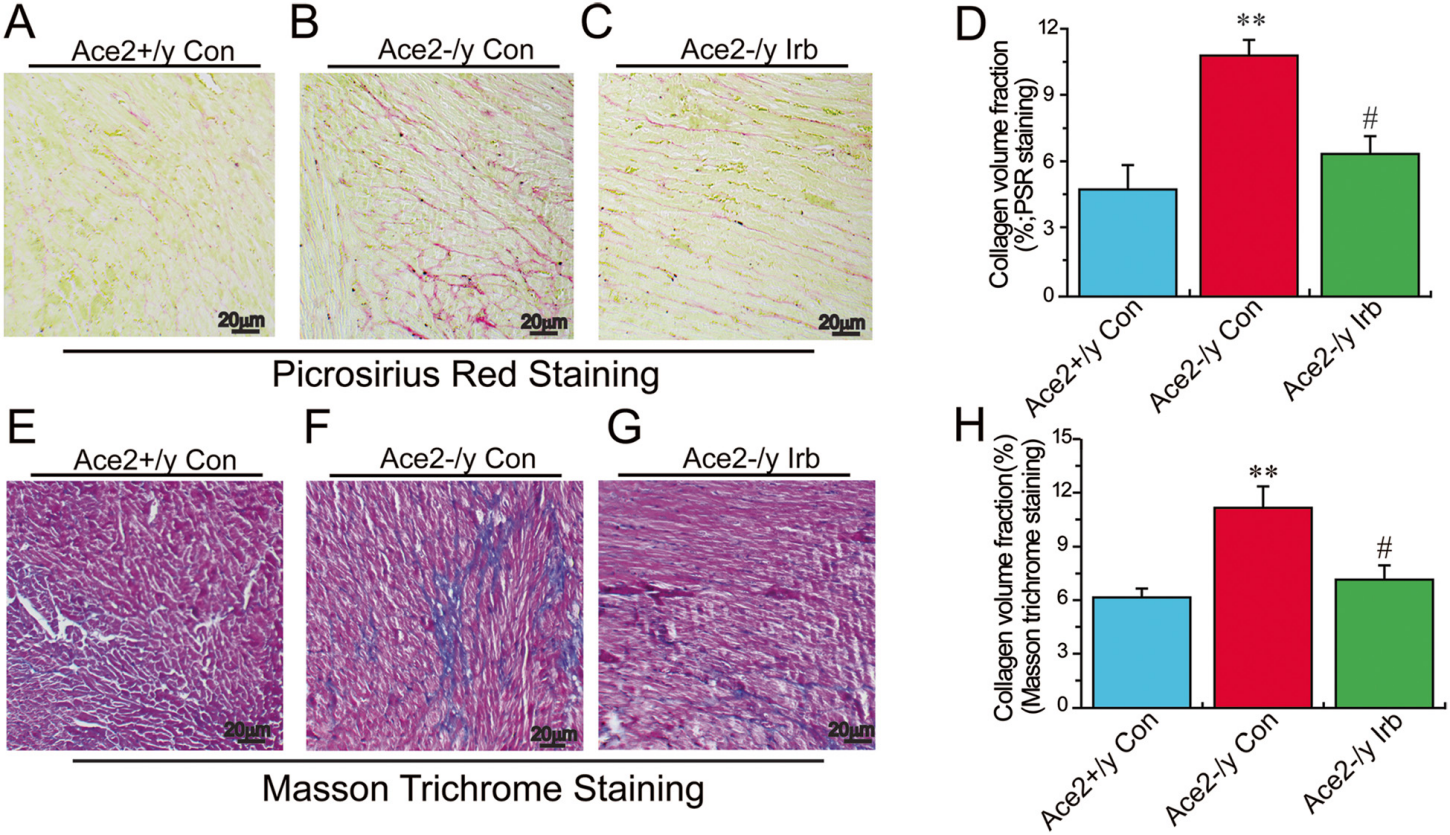
**Effects of irbesartan on myocardial fibrosis in ACE2KO mice.** Representative images of Picrosirius red **(A−D)** and Masson trichrome **(E−H)** staining showed myocardial fibrosis and collagen volume fraction (CVF) were attenuated in irbesartan (Irb)-treated Ace2^-/y^ mice than in Ace2^-/y^ control mice (×100 magnification). n = 4 for each group. **P < 0.01 compared with Ace2^+/y^ control group; #, *P* < 0.05, compared with Ace2^-/y^ control group.

**Figure 2:**
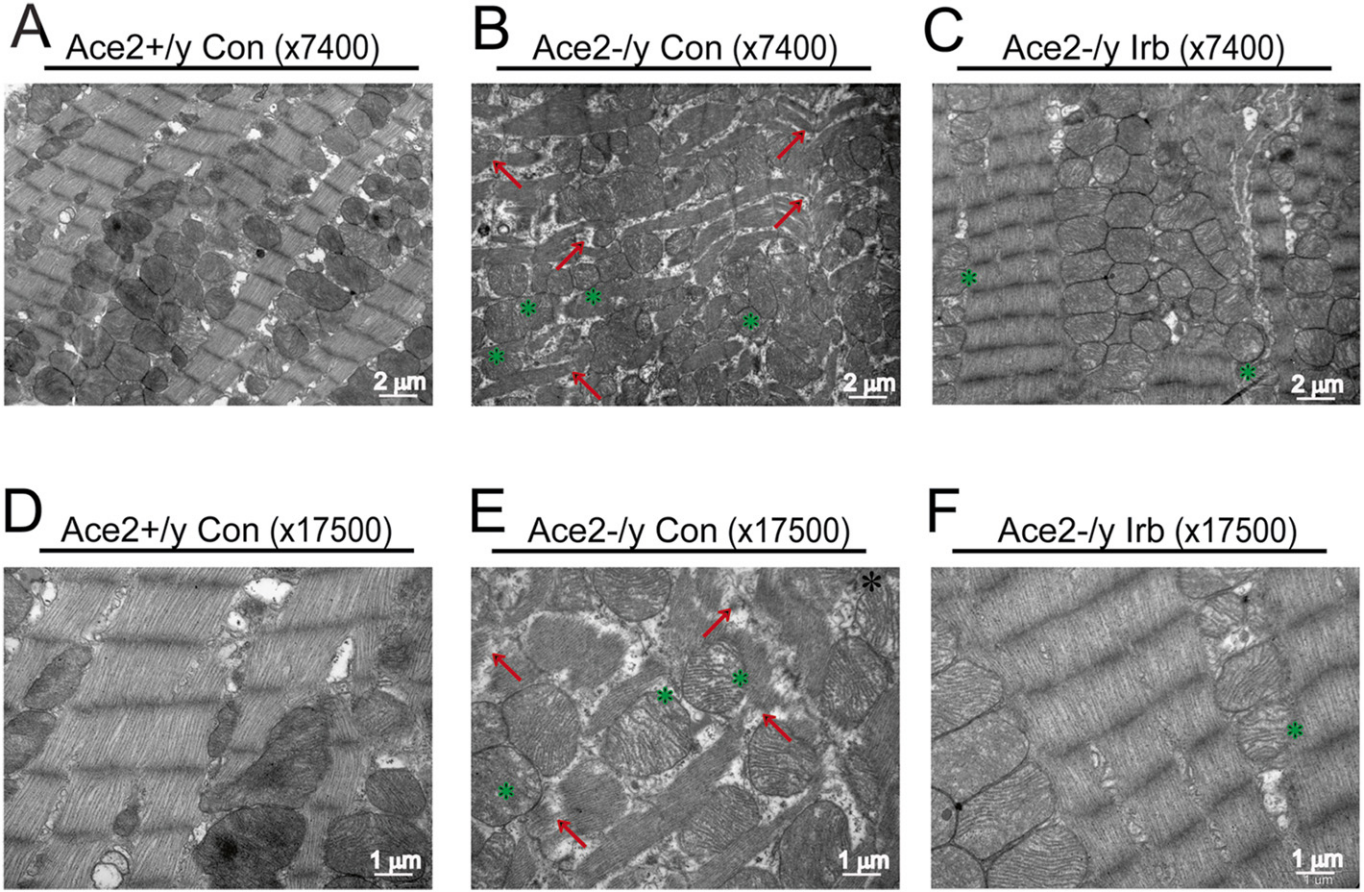
**Effects of irbesartan on myocardial ultrastructure injury in ACE2KO mice.** The myocardial ultrastructural changes were observed in Ace2^+/y^ control **(A,D)**, Ace2^-/y^ control **(B,E)** and irbesartan (Irb)-treated Ace2^-/y^ mice **(C,F)** by transmission electron microscope analysis (×7400 and × 17500 magnification). Compared with Ace2^+/y^ mice, severe myocardial ultrastructure injury was observed in Ace2^-/y^ mice, characterized with disruption or dissolution of myocardial myofilaments, myofilaments arranged irregularly and loosely (red arrow), and vacuolar degenerational and swollen mitochondria (green star).

We firstly evaluated the effects of ACE2 deficiency on myocardial fibrosis and injury. As shown in Figures 1 and 2, loss of ACE2 resulted in increased myocardial fibrosis and severe myocardial ultrastructure injury in the ACE2KO mice as assessed by PSR and Masson trichrome staining and transmission electron microscope analysis. Compared with Ace2^+/y^ mice, the myocardial collagen volume fraction (CVF) showed a marked increase in ACE2KO mice, which were significantly prevented by treatment with the AT1 blocker irbesartan (Figure 1) (n = 4; *P* < 0.05 or *P* < 0.01, respectively). In the ACE2KO hearts, myocardial myofilaments were disrupted and disarranged associated with vacuolar degenerational and swollen mitochondria (Figure 2). Notably, in response to irbesartan treatment, myocardial fibrosis and ultrastructure injury were alleviated in ACE2-null mice (Figures 1 &2).

### Treatment with irbesartan prevented ACE2 deficiency-mediated myocardial hypertrophy in ACE2KO mice with activation of the PPARγ signaling

**Figure 3:**
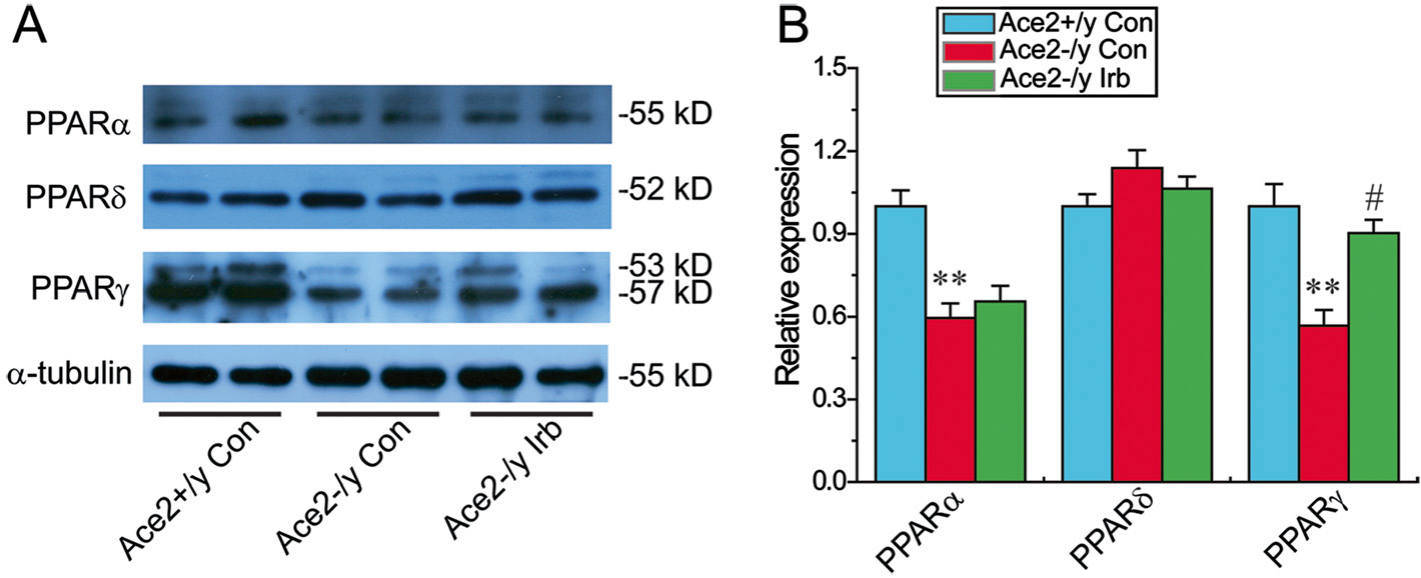
**Cardiac protein levels of PPARα, PPARδ and PPARγ in mice.** Representative Western blot **(A)** exhibited cardiac protein expression **(B)** of PPARα, PPARδ, and PPARγ in mice. α-tubulin was used as endogenous control. Irb = irbesartan, PPAR, peroxisome proliferator-activated receptor. n = 5 for each group. ^**^P < 0.01 compared with Ace2^+/y^ control group; #, *P* < 0.05 compared with Ace2^-/y^ control group.

**Figure 4:**
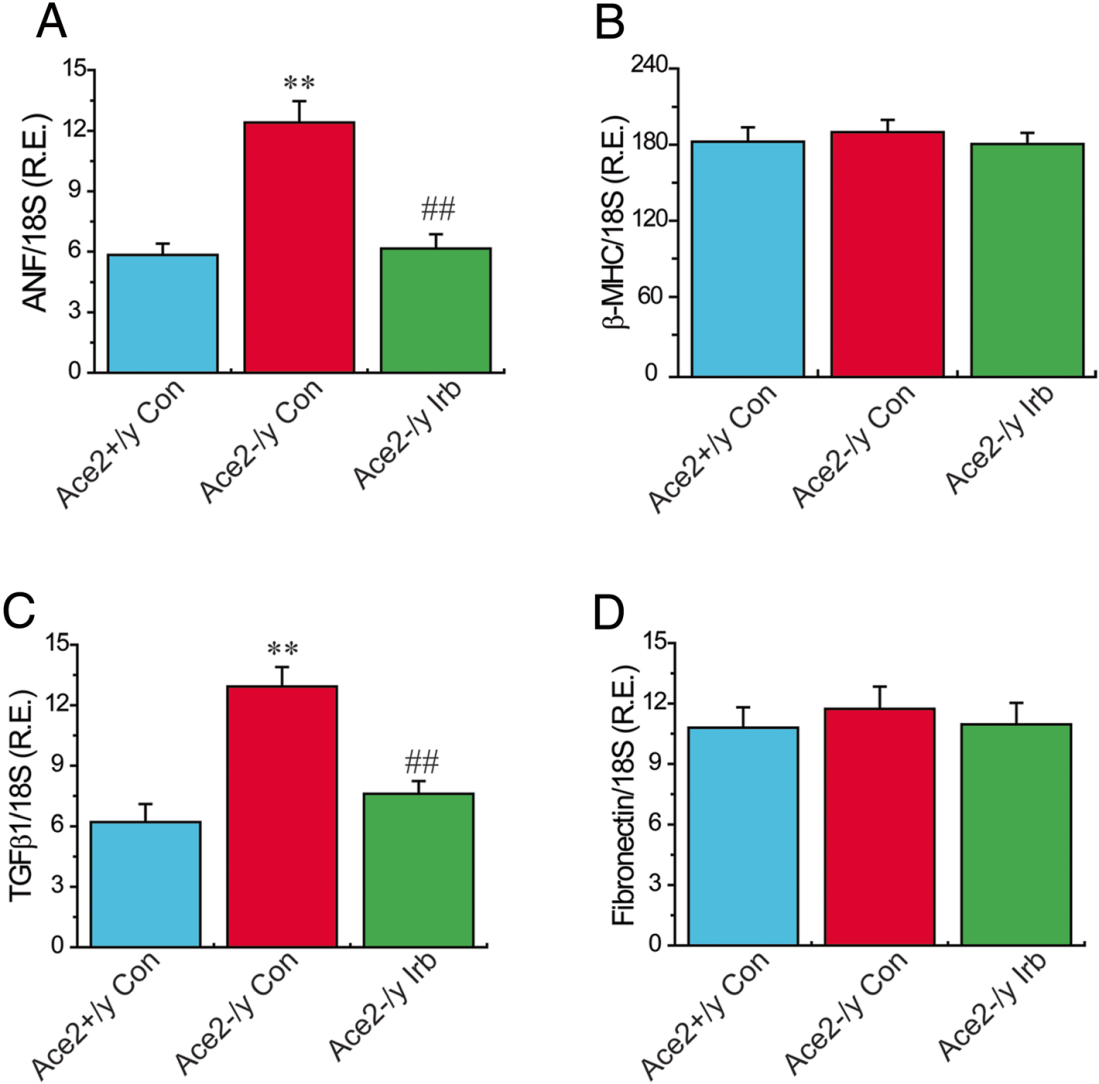
**Cardiac mRNA levels of hypertrophy and fibrosis related genes in mice.** TaqMan real-time PCR analysis revealed mRNA expression of hypertrophy and fibrosis related genes including ANF **(A)**, β-MHC **(B)**, TGFβ1 **(C)**, and fibronectin **(D)** in the hearts of mice. 18S rRNA was used as an endogenous control. R.E. = relative expression; ANF, atrial natriuretic factor; β-MHC, β-myosin heavy chain; TGFβ1, transforming growth factor-β1. n = 6 for the Ace2^+/y^ control group and Ace2^-/y^ irbesartan group; n = 8 for the Ace2^-/y^ control group. ^**^P < 0.01 compared with Ace2^+/y^ control group; ##, *P* < 0.01 compared with Ace2^-/y^ control group.

We next evaluated the regulatory roles of ACE2 deficiency on cardiac PPARs signaling and myocardial hypertrophy. As shown in Figure 3 and Table 2, loss of ACE2 resulted in marked decreases in cardiac expression of PPARα and PPARγ in ACE2KO mice (n = 3-5; *P* < 0.05 or *P* < 0.01, respectively), without affecting the expression of PPARδ. In addition, loss of ACE2 led to marked increases in left ventricular (LV) posterior wall thickness (LVPWT), LV weight (LVW) and the ratio of LVW and body weight (BW) in ACE2KO mice (Table 3) (n = 6−8; *P* < 0.05, respectively). TaqMan real-time PCR analysis revealed that there was an obvious increase in mRNA expression of hypertrophy-associated gene ANF (Figure 4A) in ACE2-deficient hearts (n = 6−8; *P* < 0.01). Echocardiographic data revealed normal systolic function with no change in LV fractional shortening and ejection fraction between WT and ACE2KO mice (Table 3). Treatment with irbesartan largely prevented ACE2 deficiency-mediated increases in LVPWT, LVW, LVW/BW ratio (Table 3) and ANF mRNA level (Figure 4A) in ACE2-null mice (n = 6−8; *P* < 0.05, respectively). However, there was no change in cardiac β-MHC mRNA level among groups (Figure 4B). Intriguingly, the AT1 blocker irbesartan significantly upregulated cardiac mRNA (Table 4) and protein (Figure 3B) levels of PPARγ in ACE2KO mice (n = 3-5; *P* < 0.05 or *P* < 0.01, respectively). However, irbesartan did not increase the expression of PPARα and PPARδ (Figure 3 & Table 4) in ACE2-deficient hearts (n = 3-5; *P* > 0.05, respectively). Our findings indicated the protective effects of irbesartan on myocardial hypertrophy in ACE2KO mice via activation of the PPARγ signaling pathway.

**Table 2:** The mRNA expression of PPAR and fibrosis-related genes in the ACE2-deficient hearts (n = 3)

**Gene name**	**Description**	**Folds up- or down-regulation (Vs. WT mice)**	***P *value**
PPARα	peroxisome proliferator activated receptor-α	−4.16	0.028
PPARδ	peroxisome proliferator activated receptor-δ	1.02	0.968
PPARγ	peroxisome proliferator activated receptor-γ	−2.06	0.004
CTGF	connective tissue growth factor	4.17	0.010
Col3A1	collagen, type III, alpha 1	2.37	0.021
FN1	fibronectin	1.18	0.635
TGF-β1	transforming growth factor-β1	2.62	0.001
TGF-β2	transforming growth factor-β2	1.21	0.542

**Table 3:** The general data in mice

	**Ace2**^**+/y **^**control**	**Ace2**^**-/y **^**control**	**Ace2**^**-/y **^**Irbesartan**
**n**	**6**	**8**	**6**
HR (bpm)	496 ± 11	494 ± 12	501 ± 10
BW (g)	26.53 ± 0.72	25.81 ± 0.61	26.68 ± 0.52
LVW (mg)	9.95 ± 0.38	12.18 ± 0.31^*^	10.35 ± 0.31
LVW/BW (mg/g)	0.37 ± 0.02	0.47 ± 0.03^*^	0.39 ± 0.02
LVEDD (mm)	3.83 ± 0.03	3.85 ± 0.03	3.86 ± 0.04
LVESD (mm)	1.75 ± 0.04	1.81 ± 0.04	1.79 ± 0.05
LVFS (%)	54.32 ± 0.93	53.05 ± 1.03	53.61 ± 1.14
LVEF (%)	62.03 ± 3.01	59.56 ± 2.37	63.83 ± 3.89
LVPWT (mm)	0.61 ± 0.03	0.78 ± 0.03^*^	0.65 ± 0.04

*HR* heart rate; *BW* body weight; *LVW* left ventricular (LV) weight; *LVEDD* LV end diastolic diameter; *LVESD* LV end systolic diameter; *LVFS* LV fractional shortening; *LVEF* LV ejection fraction; *LVPWT* LV posterior wall thickness. n = 6-8. Results are presented as mean ± SEM. ^*^, p < 0.05 compared with all other groups.

**Table 4:** The mRNA expression of PPAR and fibrosis-related genes in ACE2 deficient hearts after irbesartan treatment (n = 3)

**Gene name**	**Description**	**Folds up- or down-regulation (Vs. ACE2KO mice)**	***P *value**
PPARα	peroxisome proliferator activated receptor-α	1.46	0.655
PPARδ	peroxisome proliferator activated receptor-δ	−1.04	0.456
PPARγ	peroxisome proliferator activated receptor-γ	3.26	0.010
CTGF	connective tissue growth factor	−3.03	0.019
Col3A1	collagen, type III, alpha 1	−2.05	0.013
FN1	fibronectin	−1.55	0.107
TGF-β1	transforming growth factor-β1	−2.55	0.008
TGF-β2	transforming growth factor-β2	−1.24	0.789

### Treatment with irbesartan reversed ACE2 deficiency-mediated activation of the TGFβ−CTGF−ERK signaling in ACE2KO mice with decreased collagen level

**Figure 5:**
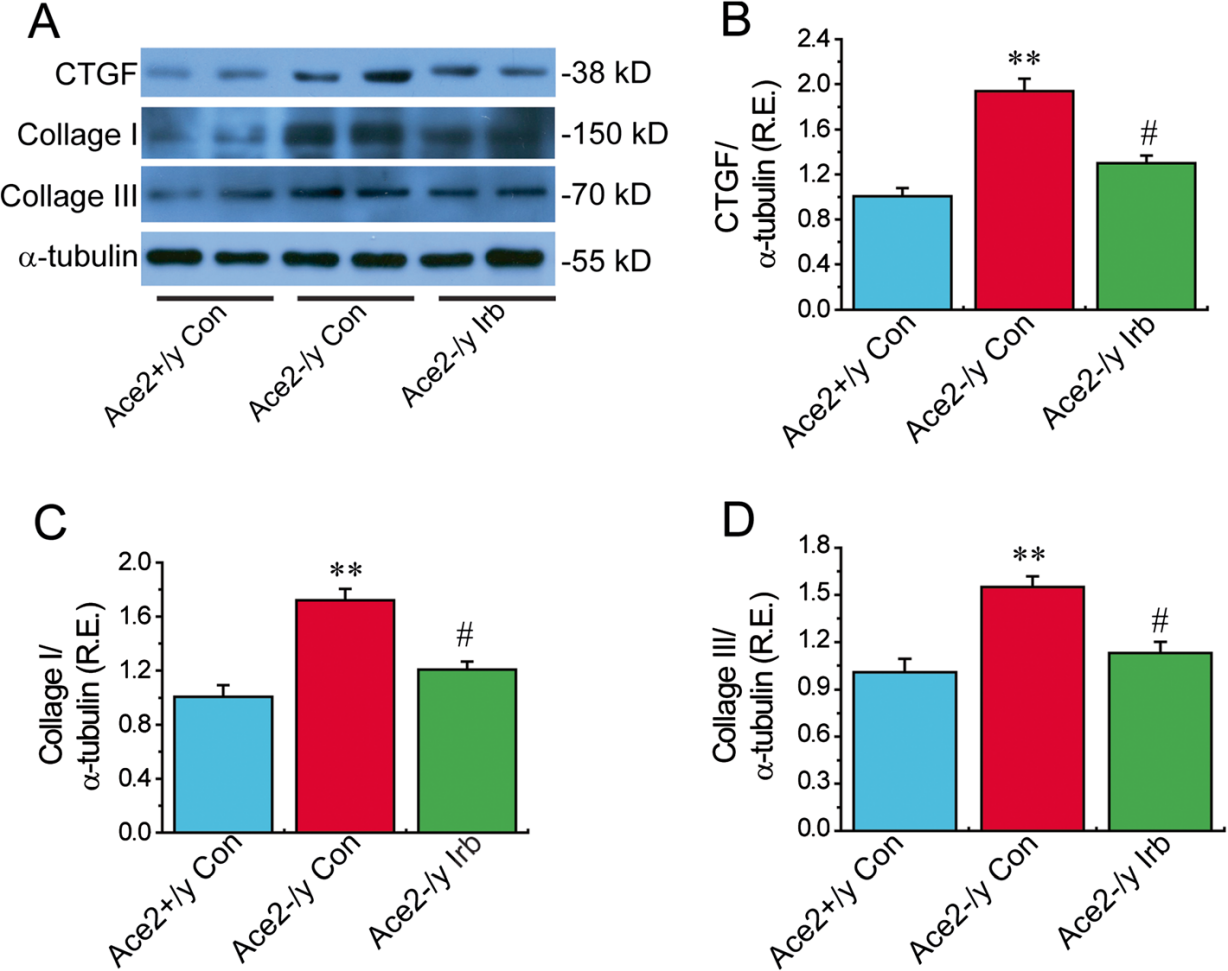
**Cardiac protein levels of CTGF and collagen in mice.** Representative Western blot **(A)** exhibited cardiac protein levels of CTGF **(B)**, collagen I **(C)** and collagen III **(D)** in mice. α-tubulin was used as endogenous control. R.E. = relative expression; CTGF, connective tissue growth factor; Irb, irbesartan. n = 5 for each group. ^**^P < 0.01 compared with Ace2^+/y^ control group; #, *P* < 0.05 compared with Ace2^-/y^ control group.

**Figure 6:**
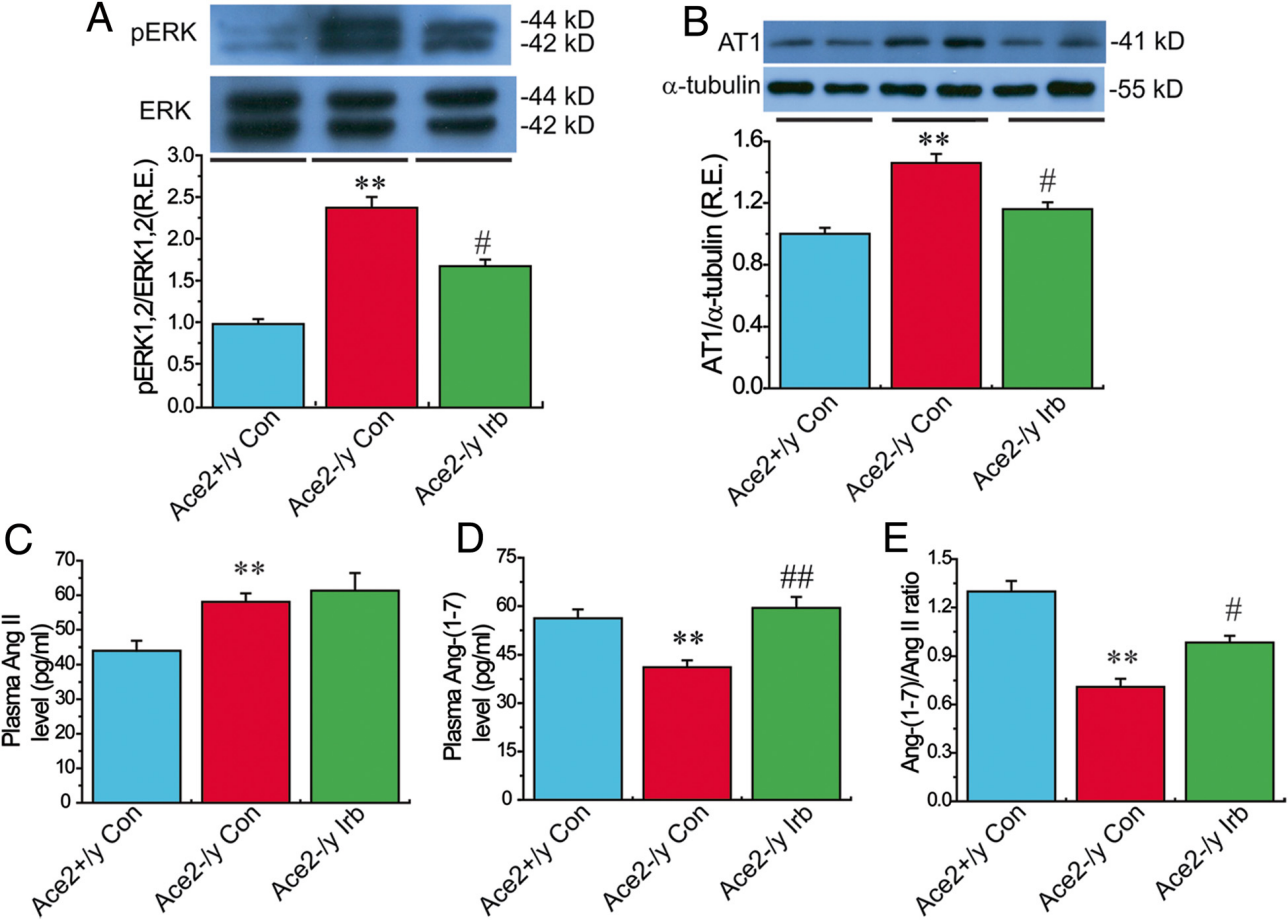
**The levels of phosphorylated-ERK1/2 (pERK1/2), AT1 receptor, Ang II and Ang-(1–7) in mice. (A−B)**, The phosphorylated level of extracellular regulated kinase1/2 (ERK1/2; **A**) and protein level of AT1 receptor **(B)** were measured by Western blotting analysis in the Ace2^+/y^ control mice and Ace2^-/y^ mice treated with irbesartan (Irb) or saline (control). n = 5 for each group. **(C−E)**, Plasma levels of Ang II **(C)** and Ang-(1−7) **(D)** and the ratio of Ang-(1−7)/Ang II **(E)** in mice. n = 6 for the Ace2^+/y^ control group and Ace2^-/y^ irbesartan group; n = 8 for the Ace2^-/y^ control group. **P < 0.01 compared with Ace2^+/y^ control group; #, *P* < 0.05, ##, P < 0.01 compared with Ace2^-/y^ control group.

We finally examined the effects of ACE2 deficiency on myocardial TGFβ−CTGF−ERK signaling and collagen production by real-time PCR gene array and Western blotting analysis. Compared with WT mice, the levels of TGFβ1, CTGF and collagen III genes were upregulated 2.62-, 4.17- and 2.37-fold in ACE2-deficient hearts (Table 2) (n = 3; *P* < 0.05 or *P* < 0.01, respectively). After irbesartan treatment, the cardiac expression of TGFβ1, CTGF and collagen III were downregulated 2.55-, 3.03- and 2.05-fold (Table 4) in ACE2KO mice (n = 3; *P* < 0.05, respectively). Increased levels of TGFβ1, CTGF and collagen III were confirmed by TaqMan real-time PCR or Western blotting analysis, which showed increases in the mRNA or protein expression of TGFβ1, CTGF and collagen III in ACE2-deficient hearts along with augmented collagen I level (Figures 4 &5) (n = 5-8; *P* < 0.01, respectively). These changes were linked with enhanced phosphorylated level of ERK1/2 (Figure 6A) in ACE2-deficient mice (n = 5; *P* < 0.01). More importantly, treatment with irbesartan significantly prevented ACE2 deficiency-induced increases in expression of TGFβ1 (Figure 4C), CTGF (Figure 5B), collagen I (Figure 5C) and collagen III (Figure 5D) and phosphorylated level of ERK1/2 (Figure 6A) in ACE2KO hearts (n = 5-8; *P* < 0.05 or *P* < 0.01, respectively). However, there were no changes in cardiac mRNA levels of TGFβ2 and FN1 among groups (Figure 4, Tables 2 &3). These changes were associated with a marked decrease in the levels of AT1 receptor and increases in plasma Ang-(1–7) level and the Ang-(1–7)/Ang II ratio in the irbesartan-treated ACE2KO mice (Figure 6) (n = 5-8; *P* < 0.05 or *P* < 0.01, respectively). These observations confirmed detrimental effects of ACE2 deficiency on myocardial fibrosis and ultrastructure injury along with reduced Ang-(1–7) level and cardiac protective effects of irbesartan through normalization of AT1 receptor and the Ang-(1–7)/Ang II ratio in ACE2-null mice.

## Discussion

In the present study, we provide the first evidence for cardiac protective roles of irbesartan in the ACE2 deficiency-mediated pathological hypertrophy, myocardial fibrosis and cardiac injury via activation of the PPARγ signaling pathway. We revealed that the deletion of the ACE2 gene resulted in pathological hypertrophy with marked increases in LVPWT, LVW and LVW/BW ratio and elevated ANF mRNA in ACE2-deficient hearts, without affecting the expression of β-MHC. These changes were linked with higher AT1 receptor level and lower Ang-(1–7) level in ACE2KO mice, which were significantly reversed by treatment with irbesartan. In addition, loss of ACE2 triggered myocardial ultrastructure injury, characterized with disruption or dissolution of myocardial myofilaments and mitochondrial vacuolar degeneration. However, there was no significant difference in systolic function between WT and ACE2KO mice on the basis of the LVEF and LVFS by echocardiography analysis. It has become clear that PPARs exist in adipose and cardiovascular tissues as ligand-activated transcription factors and pharmacological activation of PPARs mediates strong organ-protective effects, including anti-hypertrophic and anti-fibrosis effects [3,13,21]. Recent studies have showed the cross-talk action between the RAS and PPARγ in cardiovascular hypertrophy and remodeling [3,22,23]. Local RAS exists in the cardiovascular tissues, and ACE2, a negative regulator of the RAS, is present in various cell-types including the cardiofibroblasts, cardiomyocytes and coronary microcirculation [20,24,25]. ACE2 plays a critical role in the control of cardiac physiology and its altered expression is linked to major pathophysiological changes of the cardiovascular system [24,26]. To investigate the mechanism of pathological hypertrophy and myocardial injury in ACE2-dificient status, we examined the changes in cardiac PPARs signaling. In ACE2KO hearts, lack of ACE2 results in marked decreases in expression of PPARα and PPARγ, without affecting the expression of PPARδ. This is in agreement with other reports showing that cardiac PPARγ mRNA was reduced in ACE2-null mice associated with myocardial hypertrophy [8]. The PPARγ deficiency has been exhibited to develop cardiac hypertrophy in PPARγ-null mice [23]. More importantly, treatment with irbesartan remarkably attenuated ACE2-dificiency induced myocardial hypertrophy and ultrastructure injury along with augmentation of cardiac PPARγ level, without having a differential effect on the expression of PPARα and PPARδ. Therefore, we speculate that upregulation of the PPARγ signaling is, at least in part, responsible for cardio-protective effects of irbesartan in ACE2-deficient mice. A limitation of the current study was that we did not examine the influence of the PPARγ antagonist GW9662 on actions of irbesartan. Treatment with GW9662 and irbesartan simultaneously would help to strengthen the mechanistic link between the PPARγ signaling and irbesartan-mediated effects on cardiac remodeling and myocardial injury in ACE2-deficient mice.

The AT1 receptor blocker irbesartan has unique properties that enable it to partially activate the PPARγ signaling, which has been implicated in controlling cardiovascular hypertrophy and fibrosis via suppression of the TGFβ−CTGF and ERK signaling pathways [15,16,22]. Evidence is emerging that the TGFβ−CTGF−ERK signaling pathway regulates diverse cellular processes as ECM deposition and differentiation, as well as cell survival and proliferation, leading to cardiac fibrosis and structural injury [15,22,27,28]. TGFβ1 and CTGF are important mediators of the profibrotic effects of the RAS, such as the differentiation and proliferation of fibroblasts and collagen deposition, as Ang II increased the TGFβ−CTGF−ERK signaling in various cells including cultured cardiomyocytes and animal models [1,2,5,6,15,28]. In contrast, PPARγ is a well-known antifibrotic factor in the cardiovascular system [8,13,23,29]. PPARγ mutant mice developed significantly more severe cardiac fibrosis to Ang II that correlated with increased expression of profibrotic genes collagen I and collagen III [23]. The PPARγ antagonist GW9662 prevented the cardiac protective effects of irbesartan in mice with myocardial hypertrophy and interstitial fibrosis [13]. Activation of PPAR-γ has been reported to suppress cardiac expression of CTGF [16] and the phosphorylation signaling of ERK [15], thereby exerting protective effect on cardiac injury. In this study, we found that consistent with the induction of cardiac hypertrophy, loss of ACE2 led to enhanced CVF, severe myocardial fibrosis and adverse myocardial ultrastructure injury. Compared with WT mice, cardiac levels of TGFβ1, CTGF, collagen I and collagen III were higher in ACE2KO mice associated with lower PPARγ level and higher phosphorylated ERK1/2 level. This is in agreement with other reports showing that the lower expression of PPARγ was observed in ACE2-deficient hearts associated with higher mRNA levels of TGFβ1, collagen I and collagen III and cardiovascular fibrosis [8]. However, there was no change in cardiac TGFβ2 and fibronectin mRNA levels between ACE2KO and WT mice. More importantly, administration of irbesartan significantly reversed ACE2 deficiency-induced increases in CVF, TGFβ1, CTGF, collagen I, collagen III and phosphorylated ERK1/2 and remarkably attenuated myocardial fibrosis and ultrastructure injury, likely driven by the elevation in PPARγ levels in ACE2KO mice. Taken together, our data demonstrate that treatment with irbesartan reversed ACE2 deficiency-mediated cardiac fibrosis and adverse myocardial injury via activation of the PPARγ signaling and suppression of the TGFβ−CTGF−ERK signaling.

## Conclusions

In summary, loss of ACE2 enhances the susceptibility to pathological hypertrophy and myocardial fibrosis, resulting in adverse myocardial ultrastructure deterioration. In contrast, treatment with irbesartan prevents ACE2 deficiency-mediated cardiac hypertrophy and myocardial ultrastructure injury in ACE2 mutant mice with elevation of the PPARγ level and suppression of the TGFβ−CTGF−ERK signaling. These results indicate cardiac protective roles of irbesartan in the prevention of myocardial hypertrophy, fibrosis and cardiac injury via activation of the PPARγ signaling and support the notion that irbesartan functions as a partial agonist of PPARγ. Targeting ACE2 and PPARγ has potential therapeutic importance for modulating cardiac remodeling, fibrosis and myocardial injury. Future studies are required to more precisely clarify regulatory roles of ACE2 and PPARγ as biomarkers of myocardial injury and related heart diseases.

## Abbreviations

ACE2: Angiotensin-converting enzyme 2; ANF: Atrial natriuretic factor; Ang II: Angiotensin II; β-MHC: β-myosin heavy chain; BW: Body weight; CTGF: Connective tissue growth factor; ECM: Extracellular matrix; ERK1/2: Extracellular signal-regulated kinase 1/2; HR: Heart rate; KO: Knockout; LV: Left ventricular; LVPWT: Left ventricular posterior wall thickness; LVW: Left ventricular weight; LVEDD: Left ventricular end diastolic diameter; LVESD: Left ventricular end systolic diameter; LVFS: Left ventricular fractional shortening; LVEF: Left ventricular ejection fraction; PPARγ: Peroxisome proliferator-activated receptor-γ; PSR: Picro-sirius red; RAS: Renin-angiotensin system; TGFβ1: Transforming growth factor-β1.

## Competing interests

The authors declare that they have no competing interests.

## Authors’ contributions

JCZ, QHS, PJG and GYO conceived the study design, participated in the acquisition of data and drafted the manuscript. ZZZ, HYJ, BS and YLX carried out the experiments and participated in the acquisition of data, analysis and interpretation. TZ and JMP have been involved in analyzing the data and provided critical advice. LL and DLZ performed the statistical analysis and helped to draft the manuscript. All authors read and approved the final manuscript.
